# Intrahepatic sarcomatoid cholangiocarcinoma: A case report of the youngest patient on record and a review of the condition's characteristics

**DOI:** 10.3389/fsurg.2022.963952

**Published:** 2022-09-01

**Authors:** Long-Fu Xi, Yun Jin, Jiang-Tao Li

**Affiliations:** Department of Surgery, Second Affiliated Hospital, Zhejiang University School of Medicine, Hangzhou, China

**Keywords:** intrahepatic sarcomatoid cholangiocarcinoma, hepatobiliary malignancy, sarcomatoid change, immunohistochemical staining, case report

## Abstract

As a rare disease, intrahepatic sarcomatoid cholangiocarcinoma (s-CCC) represents less than 1% of malignancies of the hepatobiliary system and its main clinical symptoms include abdominal pain and fever. Results of pathological examinations, despite being the “gold standard”, can easily be confused with hepatocellular carcinoma (HCC). This report is about a 32-year-old male patient who was hospitalized due to occupancy of segment V of the liver for three days and had a history of chronic hepatitis B (CHB) over a 20-year span. Magnetic resonance imaging (MRI) showed a 43 mm × 52 mm-sized liver mass in the V segment, with patchy peripheral enhancement during the arterial phase and rapid wash-out during the portal and late phases. A laparoscopic hepatectomy of segment V, along with cholecystectomy, was performed. Histopathological and immunohistochemical examinations indicated a malignant neoplasm that was positive for vimentin and cytokeratin, with these features providing a positive diagnosis for intrahepatic sarcomatoid cholangiocarcinoma. After surgery, an adjuvant therapy of albumin-paclitaxel combined with gemcitabine regimen was given. No recurrence was found six months after the surgery, with follow-up still ongoing. This report aims to improve the awareness, diagnosis, and treatment of s-CCC.

## Introduction

Primary liver cancer (PLC) is one of the malignant tumors with high morbidity and mortality (1). There was about 906,000 patients with primary liver cancer who were diagnosed each year, and nearly 830,000 patients died of PLC annually (2). According to histopathology, PLC is divided into three types as following: hepatocellular carcinoma (HCC), intrahepatic cholangiocarcinoma (ICC) and mixed hepatocellular cholangiocarcinoma (HCC-ICC), of which HCC is the predominant one. Intrahepatic cholangiocarcinoma, which accounts for 5% to 30% of PLC, is a rare hepatobiliary malignancy that arises from the epithelial cells of the intrahepatic bile ducts. However, intrahepatic sarcomatoid cholangiocarcinoma (s-CCC) is one of extremely rare type of ICC (3).

Intrahepatic sarcomatoid cholangiocarcinoma (s-CCC) is a condition that can affect different organs of the body including the urinary tract, uterus, thyroid, breast, skin, pancreas, lung, upper digestive tract and the gall bladder (4). However, being a rare disease that represents less than 1% of the malignancies affecting the hepatobiliary system (5), its pathogenesis remains to be clarified. Indeed, even though possible links between sarcomatoid hepatocellular carcinoma (HCC) and preoperative anticancer treatments such as radiofrequency ablation, transcatheter arterial chemoembolization or percutaneous ethanol injection have been reported (6), similar associations between s-CCC and anticancer treatments are yet to be demonstrated. Intrahepatic sarcomatoid cholangiocarcinoma is also most commonly characterized by fever and abdominal pain and since it is easily confused with hepatocellular carcinoma and other liver tumors, immunohistochemical staining is required for an accurate diagnosis. Although middle-aged and elderly individuals over 40 years old are considered to be the main high-risk group of s-CCC (7), in this study, the case of a 32-year-old patient, diagnosed with the condition, is described. A discussion of relevant literature is also undertaken in view of improving the awareness, diagnosis, and treatment of s-CCC.

## Case report

The patient, a 32-year-old man was examined at a different hospital three days before and was found to have a hepatic V-segment occupancy. He complained of occasional upper abdominal pain at night, accompanied by nausea, but without fever, chills and other discomforts. With a history of chronic hepatitis B spanning over 20 years, the patient had also been under entecavir treatment. A physical examination showed no abnormal findings and no relevant family history was known.

The primary laboratory results showed normal levels for alanine aminotransferase (ALT), serum carcinoembryonic antigen (CEA), alpha-fetoprotein (AFP), serum total bilirubin and serum aspartate aminotransferase (AST). The tests also confirmed positive for hepatitis B surface antigen and hepatitis B core antibody in the serum.

**Figure 1:**
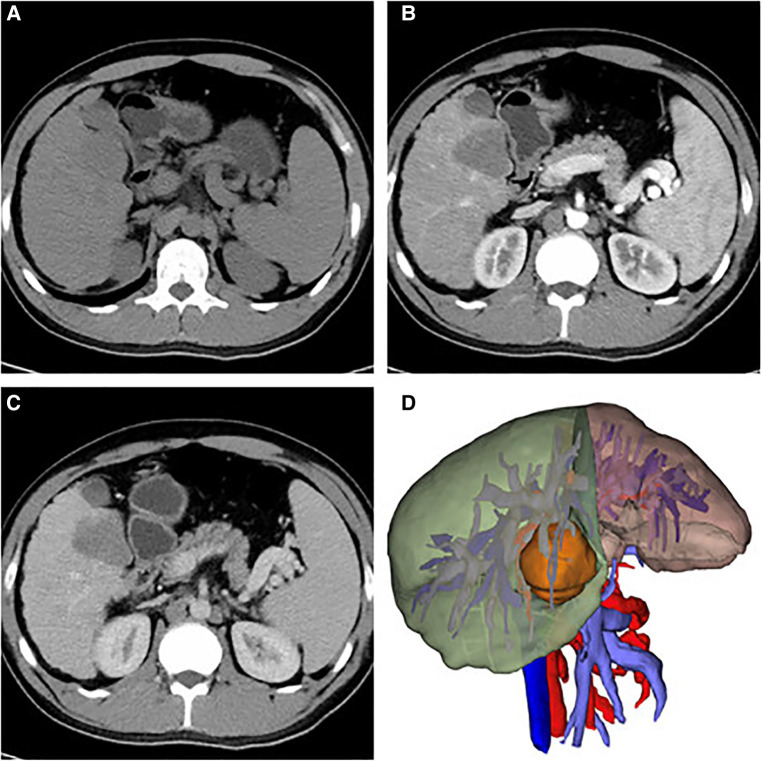
Ct images of axial sections: (**A**) A bulky hypodense mass in segment V of the liver indicates the lesion; arterial phase (**B**) shown enhancement of peripheral areas and slight progressive centripetal enhancement in portal phase (**C**); three-dimensional reconstruction of the liver mass in segment V (43 mm × 52 mm-sized) (**D**).

**Figure 2:**
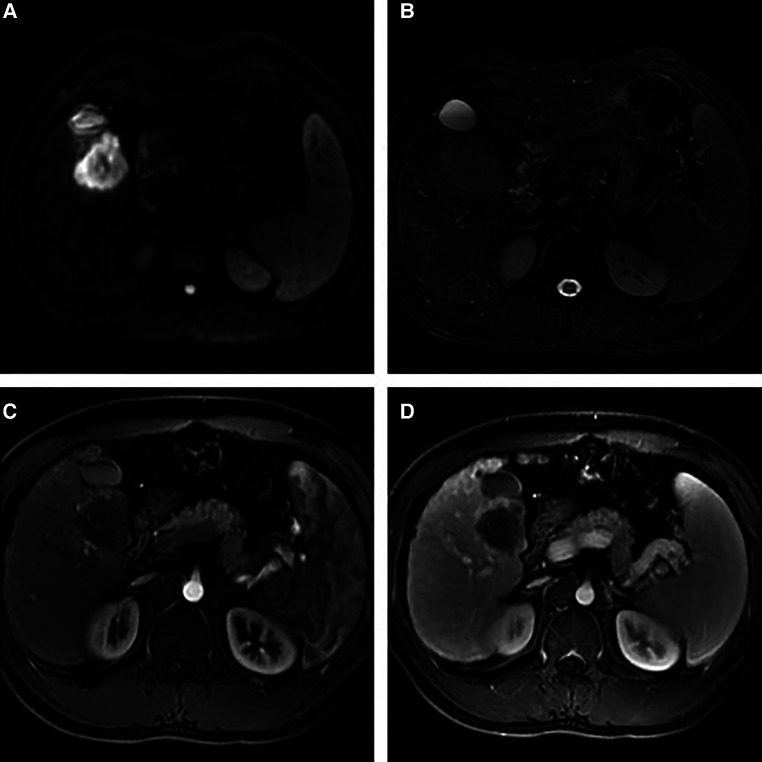
Contrasted MRI images of the liver: (**A**) an abnormal signal liver mass in segment V and obvious restricted diffusion on DWI; (**B**) a hypointense signal and a slightly hyperintense one on T1- and T2-weighted scans respectively; (**C**) patchy peripheral enhancement in the arterial phase, and (**D**) rapid washout in the portal and late phases.

A 43 mm × 52 mm-sized hypodense liver mass was observed in segment V based on contrasted computed tomography (CT) scans, with progressive peripheral enhancement during the arterial phase and a rapid wash-out during portal and late phases (Figure 1). A similarly sized mass was observed during contrasted liver magnetic resonance imaging (MRI), with a hypointense signal and a slightly hyperintense one noted on T1- and T2-weighted scans respectively. Additionally, obvious diffusion restriction was observed on diffusion weighted imaging (DWI) along with a hypointense signal in apparent diffusion coefficient (ADC) mapping, with patchy peripheral enhancement during the arterial phase and a rapid wash-out during the portal and late phases (Figure 2).

A laparoscopic segmentectomy of segment V of the liver was carried out along with cholecystectomy and no postoperative bleeding, biliary fistula or other complications occurred. Following the surgery, the patient had an uneventful recovery and was therefore discharged after eight days.

**Figure 3:**
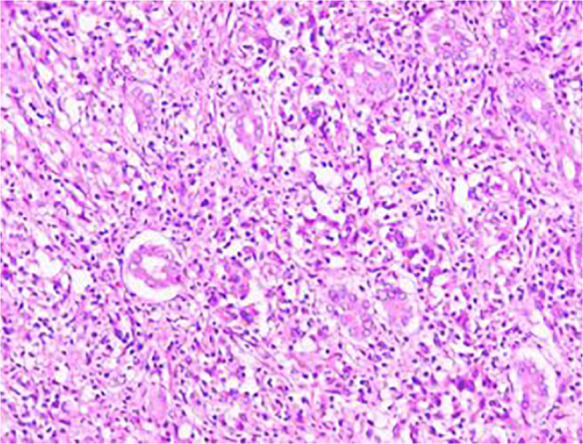
Histologic findings (hematoxylin and eosin staining, 40 × ): primary high-grade malignant tumor with patchy necrosis and abundant lymphocyte infiltration. Cancer infiltration was not observed in nerves, in vessels or at the margin of hepatectomy.

Histological examinations showed a primary high-grade malignant tumor with patchy necrosis and abundant lymphocyte infiltration. The absence of cancer infiltration in nerves, in vessels or at the margin of hepatectomy was also noted (Figure 3). Furthermore, the neoplasm was found to be positive for vimentin, programmed cell death ligand 1 (PD-L1), cytokeratin (CK) 19, CK7, cluster of differentiation (CD) 34 (vessels) and CD31 (vessels) after immunohistochemical examination while staining for hepatocyte paraffin 1 (HepPar-1), AFP, CK20, Glypican-3 returned negative results. The Ki-67 score was approximately 50%. Altogether, the above results confirmed the diagnosis for intrahepatic sarcomatoid cholangiocarcinoma. An adjuvant therapy of albumin-paclitaxel combined with gemcitabine regimen was given for 8 cycles of treatment after surgery. No recurrence was found seventeen months after the operation, with follow-up still ongoing.

## Discussion

Intrahepatic sarcomatoid cholangiocarcinoma represents a rare form of intrahepatic cholangiocarcinoma that mostly affects middle-aged and elderly individuals over 40 years old (8). This case report is about a 32-year-old man, the youngest patient with the condition.

Around 4.5% of autopsied and surgical cases of intrahepatic cholangiocarcinoma show sarcomatoid transformation (9), with similar transformations observed in sarcomatoid HCC which accounts for 3.9%–9.4% of HCC autopsy cases. As pointed out earlier, sarcomatoid HCC is reportedly linked with various preoperative anticancer treatments, including radiofrequency ablation, transcatheter arterial chemoembolization or percutaneous ethanol injection (10).

On the other hand, the pathogenesis of s-CCC has not yet been clarified, although a few possibilities have been put forward. Firstly, it was suggested to be the result of sarcomatoid transdifferentiation or the dedifferentiation of primary epithelial carcinoma cells, with these processes often referred to as metaplastic transformation or epithelial-mesenchymal transition (EMT). It is also possible that pluripotent stem cells could be undergoing biphasic differentiation to sarcoma or carcinoma in various directions, resulting in a combination of both cell types depending on the growth of each tumor type. Finally, carcinoma cells could give rise to cancer cells with multipotent differentiation potency that subsequently undergo sarcomatoid redifferentiation (5).

s-CCC can be asymptomatic, but it may also manifests itself by nonspecific symptoms or signs including weight loss, fatigue, nausea, fever and abdominal pain, with the latter two being more specific indicators of the condition. For the young patient under study, upper abdominal pain accompanied by nausea was reported but there was no fever or any other forms of discomfort.

Previously, common serological markers such as AFP, CA19–9, CEA as well as protein induced by vitamin K absence or antagonist-II (PIVKA-II), were negative or low for s-CCC (11–12). However, when analyzing the level of tumor markers in 11 cases of s-CCC, Kim D. K et al. found that they were not reliable to diagnose s-CCC (5). Similarly, the current report found the young patient's serum CEA and AFP content to be at normal levels. Moreover, even though hypoattenuation and peripheral region enhancement are common characteristics observed for s-CCC after contrast injection during CT scans, similar features may also be visible during CT scans of HCC, thus, making these features, on their own, unsuitable to distinguish s-CCC from HCC.

However, s-CCC also exhibits unique histopathological and molecular patterns, with one of these being the fact that its tumor cells usually have exact malignant epithelial cholangiocytic and sarcomatoid biphasic components (13). The cholangiocarcinomatous nature of tumors is normally diagnosed with the help of immunohistochemistry, with previous immunohistochemical results revealing that s-CCC tumors tend to be positive for both vimentin, the mesenchymal tumor marker as well as CK7 and CK8, the epithelial cholangiogenic tumor markers while tests for HepPar-1 tend to be yield negative results (14). For the current study, histological examinations showed a primary high-grade malignant tumor with patchy necrosis and abundant lymphocyte infiltration. Immunohistochemical examinations further showed positive staining for vimentin, CK19, CK7, CD34 (vessels), CD31 (vessels) and PD-L1 while results for AFP, Glypican-3, CK20, PD-L1 and HepPar-1 were negative. The latter, as a marker of hepatocytes, helps to distinguish cholangiocarcinoma and metastatic carcinoma in the liver from HCC and as such, it is of diagnostic value.

In addition to the above, the common characteristics of s-CCC were also reviewed by searching for relevant case reports or series from the available literature in PubMed (English only), with Table 1 showing the clinical aspects of 76 identified cases of s-CCC. Of the patients reported in the studies (age range: 32–87; mean age: 61 years old), 52 (68.4%) were males and 24 (31.6%) were females. The tumor size was, on average, 8.0 cm (range: 2.0–25.0 cm). The main complaint was also recorded for 52 patients, with the most common one being abdominal pain which occurred in 30 patients (57.7%). In contrast, only 15 patients (28.8%) complained of fever while 7 patients (13.5%) reported weight loss.

**Table 1:** Clinical findings and immunohistochemistry results of patients diagnosed with intrahepatic sarcomatoid cholangiocarcinoma.

Ref.	Case	Age/sex	Main complaint	Tumor size (cm)	Treatment	Histological examination	Immunohistochemical results	
							positive	negative
Malhotra S et al. (4)	1	60/F	Right upper abdominal pain, an upper abdominal mass	20.0	Operation, chemotherapy after recurrence	Sarcomatoid cholangiocarcinoma	CEA, CK19, CK7, AE1/AE3, EMA	HepPar-1
Tsou YK et al. (9)	2	77/F	Abdominal pain, a palpable mass, body weight loss	14.0	None	Intrahepatic cholangiocarcinoma with sarcomatoid changes	CK7, AE1/AE3, vimentin	HepPar-1, desmin, c-kit, S-100
	3	62/M	Abdominal pain, body weight loss	3.0	N/A	Intrahepatic cholangiocarcinoma with sarcomatoid changes	CK7, AE1/AE3, vimentin	HepPar-1, c-kit
	4	59/M	Abdominal pain, palpable mass, body weight loss	11.0	None	Intrahepatic cholangiocarcinoma with sarcomatoid changes	CK7, AE1/AE3, vimentin	HepPar-1, c-kit, S-100, CD34, actin
	5	63/M	Dyspnea, body weight loss	14.0	None	Intrahepatic cholangiocarcinoma with sarcomatoid changes	CK7, AE1/AE3, vimentin	C-kit, CD34, actin
	6	64/M	Back pain	11.0	Operation, radiotherapy for spinal metastasis	Intrahepatic cholangiocarcinoma with sarcomatoid changes	CK7, AE1/AE3, vimentin	HepPar-1, S-100, actin
	7	50/F	Abdominal pain	4.5	Operation	Intrahepatic cholangiocarcinoma with sarcomatoid changes	CK19, AE1/AE3, vimentin, CA19-9	N/A
	8	69/F	Abdominal pain, fever	2.5	Operation	Intrahepatic cholangiocarcinoma with sarcomatoid changes	CK7, vimentin	N/A
Kaibori M et al. (8)	9	69/F	fever, abdominal pain	22.0	Operation	Cholangiocarcinoma with extensive sarcomatous changes	CK, EMA, vimentin	AFP, CEA, S-100
Lim BJ et al. (11)	10	41/F	A palpable epigastric mass	17.0	Operation	A rhabdoid cholangiocarcinoma	CEA, vimentin, CK-pan	c-kit, AFP, CD34, AMA, HMB-45, S-100, CK20, CK7
Kim DK et al. (5)	11	45/M	Right upper quadrant pain	7.5	Chemotherapy	N/A	CK19, vimentin	HSA, CD10
	12	67/M	Left flank pain	2.5	Chemotherapy	N/A	AFP, CEA, vimentin, CK	CD117, c-kit, HSA, CK19, CK7
	13	55/M	Right upper quadrant pain, fever	6.5	Chemotherapy	N/A	vimentin, CK19, CK	S-100, c-kit, CEA, EMA, Desmin, CK8
	14	66/M	Right upper quadrant pain, fever	10.0	Supportive treatment	N/A	EMA, CEA, vimentin, CK19, CK8, CK	TTF-1, AFP, HSA
	15	56/M	Right upper quadrant pain, fatigue	8.0	Chemotherapy	N/A	SMA, vimentin, CK19, CK8, CK	HMW-CK, CD68, CD5, HSA
	16	66/F	Right upper quadrant pain	7.5	Chemotherapy	N/A	CEA, vimentin, CK19, CK8, CK7	HSA
	17	68/M	Weight loss, fatigue	6.0	Supportive treatment	N/A	CD34, vimentin, CK19, CK8, CK7	HMW-CK, CEA, HSA
	18	55/F	Right upper quadrant pain, fever	8.5	Chemotherapy	N/A	p53, CEA, vimentin, CK19	CD34, CD31
	19	49/M	Left upper quadrant pain, fever	9.5	Chemotherapy	N/A	CEA, vimentin, CK19	S-100, c-kit, SMA, HSA, Desmin, CK7
	20	65/M	Right upper quadrant pain	9.5	Supportive treatment	N/A	CEA, vimentin, CK19, CK	CD31, HSA
	21	61/M	Right upper quadrant pain	5.0	Viscum album	N/A	MUC1, vimentin, CK19, CK7	CD10, HSA
Sintra S et al. (7)	22	N/A, M	None	10.0	Palliative care	A malignant tumor with a carcinomatous and a sarcomatous component	CK7, vimentin	CK20, HepPar1
Sasaki M et al. (17)	23	79/M	Epigastic pain, weight loss, fever	8.0	None	Perferation of atypical fibrohistioytic spindle or giant cell	CEA, vimentin, EMA, KER	AAT, S-100, AFP,
Haratake J et al. (18)	24	59/M	Fever, icterus, an abdominal mass	Fist-sized	Supportive	Poorly differentiated adenocarcinoma	Vimentin, low molecular cytokeratin	Desmin, UEA-1
Gu KW et al. (26)	25	65/M	N/A	N/A	Radiotherapy/Chemotherapy	Favor sarcomatous ICC	N/A	N/A
	26	70/M	N/A	N/A	Operation	Sarcomatous ICC	N/A	N/A
	27	48/F	N/A	N/A	Operation	Sarcomatous ICC	N/A	N/A
	28	45/M	N/A	N/A	Chemotherapy/radiotherapy	Sarcomatous ICC	N/A	N/A
	29	46/F	N/A	N/A	Chemotherapy/radiotherapy	Favor sarcomatous ICC	N/A	N/A
	30	69/M	N/A	N/A	Operation	Sarcomatous ICC	N/A	N/A
	31	54/F	N/A	N/A	Operation	Sarcomatous ICC	N/A	N/A
	32	74/M	N/A	N/A	Operation	Sarcomatous ICC	N/A	N/A
	33	57/M	N/A	N/A	Chemotherapy/radiotherapy	Favor sarcomatous ICC	N/A	N/A
	34	51/M	N/A	N/A	Operation	Sarcomatous ICC	N/A	N/A
	35	69/M	N/A	N/A	Chemotherapy/radiotherapy	Sarcomatous ICC	N/A	N/A
	36	61/F	N/A	N/A	Operation	Sarcomatous ICC	N/A	N/A
	37	53/M	N/A	N/A	Operation	Sarcomatous ICC	N/A	N/A
	38	56/F	N/A	N/A	Operation	Sarcomatous ICC	N/A	N/A
	39	62/F	N/A	N/A	Operation	Sarcomatous ICC	N/A	N/A
	40	64/M	N/A	N/A	Operation	Sarcomatous ICC	N/A	N/A
	41	59/M	N/A	N/A	Operation	Sarcomatous ICC	N/A	N/A
	42	64/F	N/A	N/A	Operation	Sarcomatous ICC	N/A	N/A
	43	52/M	N/A	N/A	Operation	Sarcomatous ICC	N/A	N/A
	44	44/M	N/A	N/A	Operation	Sarcomatous ICC	N/A	N/A
	45	48/F	N/A	N/A	Operation	Sarcomatous carcinoma	N/A	N/A
	46	68/M	N/A	N/A	Chemotherapy/radiotherapy	Sarcomatous carcinoma	N/A	N/A
Honda M et al. (21)	47	61/F	Back pain	N/A	None	Tubular adenocarcinoma consistent with cholangiocarcinoma, and sarcomatous carcinoma	vimentin	S-100, desmin, AFP, albumin, myoglobin
Watanabe et al. (25)	48	62/M	Jaundice, A liver tumor	5.0	Operation, chemotheray after surgery	Sarcomatous ICC	CK, vimentin	N/A
Nakajima T et al. (19)	49	84/F	Anorexia, jaundice, abdominal pain	3.5	None	Moderately differentiated adenocarcinoma	CA19-9, EMA, KER	NSE, S-100, desmin, actin, vimentin, AFP, CEA, PAS
	50	43/F	Right hypochondralgia, fever	14.0	Right hepatic lobectomy	Moderately differentiated adenocarcinoma	Vimentin, EMA, KER	NSE, S-100, desmin, actin, CA199, AFP, CEA, PAS
	51	73/F	Abdominal mass	7.0	chemotherapy	Moderately differentiated adenocarcinoma	None	Vimentin, EMA, KER, NSE, S-100, desmin, actin, CA199, AFP, CEA, PAS
	52	37/M	Abdominal discomfort, epigastralgia	10.0	None	Moderately differentiated adenocarcinoma	Vimentin, EMA, KER, PAS	NSE, S-100, desmin, actin, AFP, CA199, CEA
	53	64/M	Abdominal discomfort, nausea	7.5	TAE	Poorly differentiated adenocarcinoma	EMA, KER	Vimentin, NSE, S-100, desmin, actin, CA199, AFP, CEA, PAS
	54	52/M	Right hypochondralgia	7.5	TAE	Poorly differentiated adenocarcinoma	CEA, EMA, KER, PAS	NSE, S-100, desmin, actin, AFP, CA199, vimentin
	55	69/M	Fever	10.0	Operation	Poorly differentiated adenocarcinoma	None	Vimentin, EMA, KER, NSE, S-100, desmin, actin, CA199, AFP, CEA, PAS
Imazu H et al. (20)	56	77/M	None	6.0	Operation	Choiangiocarcinoma with sarcomatous transformation	CEA, vimentin, wide-spectrum keratin	AFP, S-100, AAT, muscle actin
Itamoto T et al. (22)	57	70/M	Fatigue, fever	8.0	TACE, Operation	Moderately differentiated tubular adenocarcinoma	Vimentin, EMA, KER	S-100, desmin, actin, CA199, CEA, AFP
Matsuo S et al. (23)	58	77/F	Upper abdominal pain	7.7	Operation	Intrahepatic cholangiocarcinoma with malignant fibrous histiocytoma-like sarcomatous change	F13a, vimentin, AAT	AFP, CEA, SMA, CYT, EMA, desmin
Sato K et al. (14)	59	87/M	Ductal enzyme levels elevated	4.0	palliative care	Moderately differentiated tubular adenocarcinoma and round cell	CK19, vimentin, CD44s	b-catenin
Bilgin M et al. (24)	60	48/M	Left upper quadrant pain, fatigue	13.0	Operation	cholangiocarcinoma with sarcomatous change	N/A	N/A
Ning Z et al. (6)	61	63/M	Right upper abdominal pain	8.0	Operation	Sarcomatous ICC	S-100, SMA, vimentin, MUC1, desmin, CK19, CD34, SOX10, STAT6, AE1/AE3	N/A
Li X et al. (27)	62	64/M	Right upper abdominal pain	2.0	Operation	Sarcomatoid intrahepatic cholangiocarcinoma	Vimentin, CK8, CK-pan	HepPar-1, CK20, CK7
Inoue Y et al. (28)	63	61/M	Abdominal pain, distention	25.0	Operation	Cholangiocarcinoma with sarcomatous changes	CK7, CK19, vimentin and keratin-1	N/A
Gupta K et al. (29)	64	50/M	Jaundice	2.0	N/A	Sarcomatous cholangiocarcinoma	Cytokeratin, vimentin	N/A
Jung GO et al. (13)	65	59/M	Right upper quadrant pain, dizziness, mild fever	18.0	Operation, chemotherapy	Sarcomatoid cholangiocarcinoma	EMA, CK-pan, CEA	Vimentin, S-100
Kim HM et al. (10)	67	67/M	Right upper quadrant pain	6.0	Operation	Sarcomatoid cholangiocarcinoma with osteoclast-like giant cells	CK19, vimentin, CD68	Hepatocyte antigen, AFP
Shi DL et al. (30)	68	55/M	N/A	N/A	Operation	Sarcomatous intrahepatic cholangiocarcinoma	N/A	N/A
	69	47/M	N/A	N/A	Operation	Sarcomatous intrahepatic cholangiocarcinoma	N/A	N/A
Wang Y et al. (31)	70	43/M	Abdominal discomfort	7.0	Operation	Intrahepatic less differentiated sarcomatoid cholangiocarcinoma	AE1/AE3, CK19, CD34	HMBE-1, AFP, TTF1, CK5/6, hepatocytes, CA19
Kuwano A et al. (32)	71	87/M	Cough, fever	8.0	Supportive treatment	G-CSF-producing intrahepatic sarcomatoid cholangiocarcinoma	AE1/AE3, CK7, CK19, G-CSF	Hep par-1, AFP
Aizawa M et al. (33)	72	69/M	Fever, body weight loss	N/A	Operation	Squamous cell carcinoma	N/A	N/A
Sohda T et al. (34)	73	56/M	Consciousness disturbance	N/A	Supportive treatment	G-CSF-producing intrahepatic sarcomatoid cholangiocarcinoma	CK19, CK20, CA19-9, G-CSF, PTH-rP	antihuman hepatocyte antigen
Suzumura K et al. (35)	73	61/F	Epigastric pain, fever	15.0	Operation	G-CSF-producing intrahepatic sarcomatoid cholangiocarcinoma	G-CSF	N/A
Shinojima Y et al. (36)	74	68/F	Erythematous eruption on face, neck, legs	6.0	Operation	Intrahepatic cholangiocarcinoma	G-CSF	N/A
Takenaka M et al. (37)	75	62/F	A large mass in the liver	12.2	Chemotherapy	Intrahepatic cholangiocarcinoma with sarcomatous change producing G-CSF	CK CAM 5.2, G-CSF, CK19, vimentin, CK7	Hepatocyte paraffin-1
Our case	76	32/M	A liver occupancy	5.2	Operation, chemotherapy	Intrahepatic sarcomatoid cholangiocarcinoma	CK19, CK7, CD34 (vessels), CD31 (vessels), PD-L1, vimentin	AFP, Glypican-3, HepPar-1, CK20, PD-L1

F, female; M, male; TAE, transarterial embolization; TACE, transcatheter arterial infusion chemotherapy; HMB-45, human melanoma black 45; AMA, anti-mitochondria autoantibodies; TTF-1, thyroid transcription factor-1; CK, cytokeratin; CEA, carcinoembryonic antigen; MUC1, mucin-1; HSA, hepatocyte specific antigen; AFP, alpha-fetoprotein; c-kit, receptor tyrosine kinase; CA19-9, carbohydrate antigen 19-9; KER, keratin; CD, cluster of differentiation; PAS, periodic acid–Schiff; AE1/AE3, pancytokeratin AE1/AE3; AE1/AE3=CK-pan; UEA-1, ulex europaeus agglutinin-1; EMA, epithelial membrane antigen; F13a, factor XIIIa; CYT, cytochrome; PTH-rP, parathyroid hormone-related protein; HMW-CK, high molecular weight cytokeratin; SMA, smooth muscle actin; NSE, neuron specific enolase; AAT, A-1-antitrypsin; STAT-6, signal transducer and activator of transcription 6; HBME-1,human bone marrow endothelial cell-1; HepPar-1, hepatocyte paraffin-1; PD-L1, programmed cell death ligand 1; G-CSF, granulocyte-colony-stimulating factor; ICC, intrahepatic cholangiocarcinoma; N/A, not available.

As far as laboratory findings were concerned, results for the level of serum aspartate aminotransferase (AST) was available for 25 patients and they ranged from 16 to 205 (median: 34, normal: <40) U/L. Similarly, for 24 patients, the level of alanine aminotransferase (ALT) ranged from 10 to 356 (median: 33.5, normal: <40) U/L. In addition, the levels of alpha-fetoprotein (AFP), carbohydrate antigen 19-9 (CA19-9) and serum carcinoembryonic antigen (CEA) were found to be high in 8 (14.5%), 22 (40.7%) and 10 (28.6%) patients respectively while they were found to be normal for 47 (85.5%), 32 (59.3%) and 25 (71.4%) patients respectively (Supplementary Table S2).

The results of immunohistochemical analyses for 50 patients were also available and are presented in Table 1. Basically, 21 patients (42.0%) were positive for CK19 while for vimentin, 38 patients (76.0%) showed positive results. Furthermore, HepPar-1 and AFP were negative for 10 (20.0%) and 18 (36.0%) patients respectively, with these markers being significantly helpful in diagnosing s-CCC.

The treatment of tumors was recorded for 74 patients (Table 1). Of these, 35 (47.3%) underwent surgery, 15 (20.3%) were given radiotherapy or chemotherapy, 8 (10.8%) underwent supportive treatment or palliative care, 5 (6.8%) had surgery followed by chemotherapy/radiotherapy, 2 (2.7%) had transcatheter arterial embolization (TAE), 1 (1.3%) received viscum album treatment and 1 (1.3%) had transcatheter arterial infusion chemotherapy (TACE) as well as surgical treatment. 7 patients (9.5%) also remained untreated.

Follow-up information was available for 73 patients (Supplementary Table S1), of whom 44 (60.3%) experienced disease progression and died while 15 (20.5%) survived. In addition, 12 patients (16.4%) also experienced disease recurrence, and 2 (2.7%) were lost during follow-up.

Electron microscopy is also used to diagnose neoplasms of s-CCC and in this case, irrespective of their hepatocyte origin, the tumors are characterized by the presence of microvilli and basement membranes (4). Hence, based on results from electron microscopy, immunohistochemistry as well as histological examinations, sarcomatoid cholangiocarcinoma can be accurately diagnosed.

Guidelines to determine the progression of s-CCC and survival of patients are yet to be clearly defined or established. Furthermore, the extent to which radiation therapy or chemotherapy can be useful against s-CCC remains to be investigated. Nevertheless, it has been reported that viscum album, an extract from a hemiparasitic plant, could exhibit positive results against s-CCC. This extract can also be useful as part of palliative therapies for terminally-ill patients who turn resistant to conventional chemotherapy, who are suffering from hematologic diseases and even for those with lung cancer or accompanying malignant pleural effusion (15, 16). With an established treatment regimen yet to be developed, surgical resection remains the preferred approach, especially since it can even be complemented with adjuvant chemotherapy for improving the survival rate. In the current report, laparoscopic segmentectomy of segment V of the liver was undertaken alongside cholecystectomy and this was followed by adjuvant therapy with albumin-paclitaxel combined with gemcitabine. No recurrence was found seventeen months after surgery, with follow-up still ongoing.

## Conclusion

Cholangiocarcinoma, with sarcomatoid features, is a rare but aggressive form of malignant tumor that can only be diagnosed through a combination of histopathological examination, electron microscopy and immunohistochemical studies. The early detection and complete removal of tumors offer the possibility for increased survival. However, in comparison with intrahepatic cholangiocarcinoma, more thorough postoperative follow-up is required for identifying recurrences or metastases.

## Data Availability

The original contributions presented in the study are included in the article/**Supplementary Material**, further inquiries can be directed to the corresponding author/s.
