# Supplementary material for: Intrahepatic sarcomatoid cholangiocarcinoma: A case report of the youngest patient on record and a review of the condition's characteristics
Source: Front Surg. 2022 Sep 1;9:963952. doi: 10.3389/fsurg.2022.963952 (PMC9476318; doi:10.3389/fsurg.2022.963952)
Supplement: Supplementary file 1 [file Data_Sheet_1.docx]

Supplementary table 1. Clinical findings of patients with intrahepatic sarcomatoid cholangiocarcinoma

| Ref. | Case | Age/sex | Chief complaint | Tumor size (cm) | Treatment | Follow-up duration (d) | Outcome |
| --- | --- | --- | --- | --- | --- | --- | --- |
| Malhotra S et al.^1^ | 1 | 60/F | Right upper abdominal pain, an upper abdominal mass | 20.0 | Operation, chemotherapy after recurrence | 870 | Alive |
| Tsou YK et al.^6^ | 2 | 77/F | Abdominal pain, a palpable mass, body weight loss | 14.0 | None | 60 | Died |
|  | 3 | 62/M | Abdominal pain, body weight loss | 3.0 | N/A | N/A | follow-up lost |
|  | 4 | 59/M | Abdominal pain, palpable mass, body weight loss | 11.0 | None | 30 | Died |
|  | 5 | 63/M | Dyspnea, body weight loss | 14.0 | None | 7 | Died |
|  | 6 | 64/M | Back pain | 11.0 | Operation, radiotherapy for spinal metastasis | 60 | Died |
|  | 7 | 50/F | Abdominal pain | 4.5 | Operation | 60 | Died |
|  | 8 | 69/F | Abdominal pain,  fever | 2.5 | Operation | 1440 | Alive |
| Kaibori M et al.^5^ | 9 | 69/F | fever, abdominal pain | 22.0 | Operation | 90 | Died |
| Lim BJ et al.^8^ | 10 | 41/F | A palpable epigastric mass | 17.0 | Operation | 60 | Alive |
| Kim DK et al.^2^ | 11 | 45/M | Right upper quadrant pain | 7.5 | Chemotherapy | 47 | Died |
|  | 12 | 67/M | Left flank pain | 2.5 | Chemotherapy | 148 | Died |
|  | 13 | 55/M | Right upper quadrant pain, fever | 6.5 | Chemotherapy | 129 | Died |
|  | 14 | 66/M | Right upper quadrant  pain, fever | 10.0 | Supportive treatment | 20 | Died |
|  | 15 | 56/M | Right upper quadrant  pain, fatigue | 8.0 | Chemotherapy | 72 | Died |
|  | 16 | 66/F | Right upper quadrant pain | 7.5 | Chemotherapy | 125 | Died |
|  | 17 | 68/M | Weight loss, fatigue | 6.0 | Supportive treatment | 19 | Died |
|  | 18 | 55/F | Right upper quadrant pain, fever | 8.5 | Chemotherapy | 31 | Died |
|  | 19 | 49/M | Left upper quadrant pain, fever | 9.5 | Chemotherapy | 43 | N/A |
|  | 20 | 65/M | Right upper quadrant pain | 9.5 | Supportive treatment | 14 | Died |
|  | 21 | 61/M | Right upper quadrant pain | 5.0 | Viscum album | 379 | Alive |
| Sintra S et al.^4^ | 22 | N/A, M | None | 10.0 | Palliative care | 45 | Died |
| Sasaki M et al.^14^ | 23 | 79/M | Epigastic pain, weight loss, fever | 8.0 | None | N/A | Died |
| Haratake J et al.^15^ | 24 | 59/M | Fever, icterus, an abdominal mass | Fist-sized | Supportive | 26 | Died |
| Gu KW et al.^23^ | 25 | 65/M | N/A | N/A | Chemotherapy/  radiotherapy | 90 | Progression |
|  | 26 | 70/M | N/A | N/A | Operation | 90 | Recurrence |
|  | 27 | 48/F | N/A | N/A | Operation | 1050 | Recurrence |
|  | 28 | 45/M | N/A | N/A | Chemotherapy/  radiotherapy | 153 | Progression |
|  | 29 | 46/F | N/A | N/A | Chemotherapy/  radiotherapy | 61 | Progression |
|  | 30 | 69/M | N/A | N/A | Operation | 30 | Recurrence |
|  | 31 | 54/F | N/A | N/A | Operation | 780 | Recurrence |
|  | 32 | 74/M | N/A | N/A | Operation | 365 | Recurrence |
|  | 33 | 57/M | N/A | N/A | Chemotherapy/  radiotherapy | 61 | Progression |
|  | 34 | 51/M | N/A | N/A | Operation | 92 | Recurrence |
|  | 35 | 69/M | N/A | N/A | Chemotherapy/  radiotherapy | 61 | Progression |
|  | 36 | 61/F | N/A | N/A | Operation | 122 | Recurrence |
|  | 37 | 53/M | N/A | N/A | Operation | 92 | Recurrence |
|  | 38 | 56/F | N/A | N/A | Operation | 61 | Recurrence |
|  | 39 | 62/F | N/A | N/A | Operation | 780 | Free |
|  | 40 | 64/M | N/A | N/A | Operation | 570 | Free |
|  | 41 | 59/M | N/A | N/A | Operation | N/A | Follow-up lost |
|  | 42 | 64/F | N/A | N/A | Operation | 600 | Free |
|  | 43 | 52/M | N/A | N/A | Operation | 61 | Recurrence |
|  | 44 | 44/M | N/A | N/A | Operation | 730 | Recurrence |
|  | 45 | 48/F | N/A | N/A | Operation | 30 | Recurrence |
|  | 46 | 68/M | N/A | N/A | Chemotherapy/  radiotherapy | 92 | Progression |
| Honda M et al.^18^ | 47 | 61/F | Back pain | N/A | None | 115 | Died |
| Watanabe et al.^22^ | 48 | 62/M | A liver tumor, jaundice | 5.0 | Operation, chemotherapy after surgery | 330 | Died |
| Nakajima T et al.^16^ | 49 | 84/F | Anorexia, jaundice, abdominal pain | 3.5 | None | 90 | Died |
|  | 50 | 43/F | Right hypochondralgia,  fever | 14.0 | Right hepatic lobectomy | 135 | Died |
|  | 51 | 73/F | Abdominal mass | 7.0 | chemotherapy | 150 | Died |
|  | 52 | 37/M | Abdominal discomfort,  epigastralgia | 10.0 | None | 75 | Died |
|  | 53 | 64/M | Abdominal discomfort, nausea | 7.5 | TAE | 30 | Died |
|  | 54 | 52/M | Right hypochondralgia | 7.5 | TAE | 60 | Died |
|  | 55 | 69/M | Fever | 10.0 | Operation | 1095 | Alive |
| Imazu H et al.^17^ | 56 | 77/M | None | 6.0 | Operation | 336 | Alive |
| Itamoto T et al.^19^ | 57 | 70/M | Fatigue, fever | 8.0 | TACE, Operation | 275 | Alive |
| Matsuo S et al.^20^ | 58 | 77/F | Upper abdominal pain | 7.7 | Operation | 150 | Died |
| Sato K et al.^11^ | 59 | 87/M | Ductal enzyme levels elevated | 4.0 | palliative care | 90 | Died |
| Bilgin M et al.^21^ | 60 | 48/M | Left upper quadrant pain, fatigue | 13.0 | Operation | 365 | Alive |
| Ning Z et al.^3^ | 61 | 63/M | Right upper abdominal pain | 8.0 | Operation | 122 | Alive |
| Li X et al.^24^ | 62 | 64/M | Right upper abdominal pain | 2.0 | Operation | 90 | Died |
| Inoue Y et al.^25^ | 63 | 61/M | Abdominal pain,  distention | 25.0 | Operation | 34 | Died |
| Gupta K et al.^26^ | 64 | 50/M | Jaundice | 2.0 | N/A | NA | N/A |
| Jung GO et al.^10^ | 65 | 59/M | Right upper quadrant pain, dizziness, mild fever | 18.0 | Operation, chemotherapy | 240 | Alive |
| Kim HM et al.^7^ | 67 | 67/M | Right upper quadrant pain | 6.0 | Operation | 180 | Alive |
| Shi DL et al.^27^ | 68 | 55/M | N/A | N/A | Operation | 61 | Died |
|  | 69 | 47/M | N/A | N/A | Operation | 180 | Died |
| Wang Y et al.^28^ | 70 | 43/M | Abdominal discomfort | 7.0 | Operation | 75 | Died |
| Kuwano A et al.^29^ | 71 | 87/M | Cough, fever | 8.0 | Supportive treatment | 20 | Died |
| Aizawa M et al.^30^ | 72 | 69/M | Fever, body weight loss | N/A | Operation | 60 | Died |
| Sohda T et al.^31^ | 73 | 56/M | Consciousness disturbance | N/A | Supportive treatment | 5 | Died |
| Suzumura K et al.^32^ | 73 | 61/F | Epigastric pain, fever | 15.0 | Operation | 90 | Died |
| Shinojima Y et al.^33^ | 74 | 68/F | Erythematous eruption on face, neck, legs | 6.0 | Operation | N/A | N/A |
| Takenaka M et al.^34^ | 75 | 62/F | A large mass in the liver | 12.2 | Chemotherapy | 90 | Died |
| Our case | 76 | 32/M | A liver occupancy | 5.2 | Operation, chemotherapy | 120 | Alive |

Abbreviations: F: female; M: male; TAE: transarterial embolization; TACE: transcatheter arterial infusion chemotherapy; N/A: not available.

Supplementary table 2. Laboratory findings of patients with intrahepatic sarcomatoid cholangiocarcinoma

| case | CEA(ng/mL) | CA19-9(U/mL) | AFP(ng/mL) | PIVKA-II(mAU/mL) | AST(U/L) | ALT(U/L) |
| --- | --- | --- | --- | --- | --- | --- |
| 1 | N/A | N/A | N/A | N/A | N/A | N/A |
| 2 | N/A | N/A | N/A | N/A | N/A | N/A |
| 3 | N/A | N/A | Normal | N/A | N/A | N/A |
| 4 | 9.00 | 4920.00 | Normal | N/A | N/A | N/A |
| 5 | N/A | N/A | Normal | N/A | N/A | N/A |
| 6 | 10.00 | N/A | Normal | N/A | N/A | N/A |
| 7 | N/A | 51.00 | Normal | N/A | N/A | N/A |
| 8 | N/A | N/A | Normal | N/A | N/A | N/A |
| 9 | Normal | 3665.00 | Normal | N/A | N/A | N/A |
| 10 | Normal | N/A | Normal | N/A | N/A | N/A |
| 11 | 0.74 | > 1200.00 | 131.67 | 69 | 25 | 19 |
| 12 | 1.45 | 3.38 | 66.45 | 16 | 31 | 10 |
| 13 | 0.10 | 3.00 | 2.54 | 35 | 54 | 96 |
| 14 | 2.35 | 1809.57 | 1.73 | N/A | 42 | 30 |
| 15 | 1.81 | 2.33 | 2.31 | 20 | 43 | 57 |
| 16 | 12.70 | 710.38 | 3.92 | N/A | 23 | 39 |
| 17 | 1.18 | 12.59 | 2.70 | 20 | 23 | 16 |
| 18 | 3.15 | > 1200.00 | 1.71 | N/A | 30 | 31 |
| 19 | 1.08 | < 2.00 | 1.52 | N/A | 80 | 30 |
| 20 | 3.56 | 599.14 | 1.02 | N/A | 37 | 47 |
| 21 | 1.81 | 5.77 | 3.02 | 14 | 34 | 36 |
| 22 | Normal | Normal | 1753 | N/A | 20 | 15 |
| 23 | Normal | Normal | Normal | N/A | 34 | N/A |
| 24 | N/A | N/A | N/A | N/A | 75 | 46 |
| 25 | N/A | 11.25 | 3.60 | N/A | N/A | N/A |
| 26 | N/A | 22.44 | N/A | N/A | N/A | N/A |
| 27 | N/A | 7.28 | 1.80 | N/A | N/A | N/A |
| 28 | N/A | 10384.00 | 2.80 | N/A | N/A | N/A |
| 29 | N/A | N/A | 1.90 | N/A | N/A | N/A |
| 30 | N/A | 25.81 | 2.70 | N/A | N/A | N/A |
| 31 | N/A | 11.34 | 1.60 | N/A | N/A | N/A |
| 32 | N/A | 6.07 | 1.80 | N/A | N/A | N/A |
| 33 | N/A | 2.00 | 6.17 | N/A | N/A | N/A |
| 34 | N/A | 11.71 | 5.10 | N/A | N/A | N/A |
| 35 | N/A | N/A | N/A | N/A | N/A | N/A |
| 36 | N/A | 886.51 | 1.60 | N/A | N/A | N/A |
| 37 | N/A | 10.55 | 93.80 | N/A | N/A | N/A |
| 38 | N/A | 1.00 | 4.30 | N/A | N/A | N/A |
| 39 | N/A | 1.81 | 35.60 | N/A | N/A | N/A |
| 40 | N/A | 11.51 | 814.80 | N/A | N/A | N/A |
| 41 | N/A | N/A | 2.70 | N/A | N/A | N/A |
| 42 | N/A | N/A | 1.30 | N/A | N/A | N/A |
| 43 | N/A | 20.73 | 3.10 | N/A | N/A | N/A |
| 44 | N/A | 6.64 | 11.10 | N/A | N/A | N/A |
| 45 | N/A | 19.81 | 1.50 | N/A | N/A | N/A |
| 46 | N/A | 8.72 | 13.70 | N/A | N/A | N/A |
| 47 | 9.00 | 13394.00 | < 10.00 | N/A | 22 | 30 |
| 48 | 1.40 | 1109.90 | N/A | N/A | 174 | 356 |
| 49 | N/A | N/A | N/A | N/A | N/A | N/A |
| 50 | N/A | N/A | N/A | N/A | N/A | N/A |
| 51 | N/A | N/A | N/A | N/A | N/A | N/A |
| 52 | N/A | N/A | N/A | N/A | N/A | N/A |
| 53 | N/A | N/A | N/A | N/A | N/A | N/A |
| 54 | N/A | N/A | N/A | N/A | N/A | N/A |
| 55 | N/A | N/A | N/A | N/A | N/A | N/A |
| 56 | <0.50 | 17.00 | 4.20 | N/A | 33 | 27 |
| 57 | Normal | 2634.00 | 293.00 | N/A | Normal | 348 |
| 58 | Normal | Normal | Normal | N/A | N/A | N/A |
| 59 | 16.20 | 2894.00 | Normal | N/A | N/A | N/A |
| 60 | N/A | 39.00 | N/A | N/A | 152 | 45 |
| 61 | 2.17 | 100.50 | 2.20 | N/A | 25 | 19 |
| 62 | Normal | 351.74 | Normal | N/A | Normal | Normal |
| 63 | 1.20 | 5.00 | N/A | N/A | 35 | N/A |
| 64 | N/A | N/A | N/A | N/A | N/A | N/A |
| 65 | 7.32 | 34.90 | 2.58 | N/A | 205 | 325 |
| 66 | 109.60 | 1598.00 | N/A | N/A | 16 | 22 |
| 67 | N/A | <27.00 | <7.00 | N/A | N/A | N/A |
| 68 | N/A | >27.00 | <7.00 | N/A | N/A | N/A |
| 69 | 1.02 | 19.90 | 66.91 | N/A | N/A | N/A |
| 70 | 2.70 | 122.2 | 2.30 | 26 | 120 | 96 |
| 71 | N/A | N/A | N/A | N/A | N/A | N/A |
| 72 | 5.20 | 1807.00 | 2.10 | N/A | 34 | 25 |
| 73 | Normal | Normal | N/A | N/A | 66 | 67 |
| 74 | 47.60 | 4293.00 | N/A | N/A | N/A | N/A |
| 75 | 227.00 | 5983.70 | N/A | N/A | N/A | N/A |
| 76 | Normal | N/A | Normal | N/A | Normal | Normal |

Abbreviations: CEA: carcinoembryonic antigen; CA19-9: carbohydrate antigen 19-9; AFP: alpha-fetoprotein; PIVKA-II: protein induced by vitamin K absence or antagonist-II; AST: aspartate aminotransferase; ALT: alanine aminotransferase; N/A: not available.

Supplementary table 3. Immunohistochemistry of patients with intrahepatic sarcomatoid cholangiocarcinoma

| case | Specimen | Histological examination | Immunohistochemical results | |
| --- | --- | --- | --- | --- |
|  |  |  | positive | negative |
| 1 | Resection | Sarcomatoid cholangiocarcinoma | EMA, AE1/AE3, CK7, CK19, CEA | HepPar-1 |
| 2 | Biopsy | Intrahepatic cholangiocarcinoma with sarcomatoid changes | CK7, AE1/AE3,  vimentin | HepPar-1, desmin, c-kit, S-100 |
| 3 | Biopsy | Intrahepatic cholangiocarcinoma with sarcomatoid changes | CK7, AE1/AE3, vimentin | HepPar-1, c-kit |
| 4 | Biopsy | Intrahepatic cholangiocarcinoma with sarcomatoid changes | CK7, AE1/AE3, vimentin | HepPar-1, c-kit, S-100, CD34, actin |
| 5 | Biopsy | Intrahepatic cholangiocarcinoma with sarcomatoid changes | CK7, AE1/AE3, vimentin | C-kit, CD34, actin |
| 6 | Resection | Intrahepatic cholangiocarcinoma with sarcomatoid changes | CK7, AE1/AE3, vimentin | HepPar-1, S-100, actin |
| 7 | Resection | Intrahepatic cholangiocarcinoma with sarcomatoid changes | CK19, AE1/AE3, vimentin, CA19-9 | N/A |
| 8 | Resection | Intrahepatic cholangiocarcinoma with sarcomatoid changes | CK7, vimentin | N/A |
| 9 | Resection | Cholangiocarcinoma with extensive sarcomatous changes | vimentin, EMA, CK | S-100, CEA, AFP |
| 10 | Resection | A rhabdoid cholangiocarcinoma | CK-pan, vimentin, CEA | CK7, CK20, S-100, HMB-45, AMA, CD34, AFP, c-kit |
| 11 | Biopsy | N/A | CK19, vimentin | HSA, CD10 |
| 12 | Biopsy | N/A | CK, vimentin, CEA, AFP | CK7, CK19, HSA, c-kit, CD117 |
| 13 | Biopsy | N/A | CK, CK19, vimentin | CK8, Desmin, EMA, CEA, c-kit, S-100 |
| 14 | Biopsy | N/A | CK, CK8, CK19, vimentin, CEA, EMA | HSA, AFP, TTF-1 |
| 15 | Biopsy | N/A | CK, CK8, CK19, vimentin, SMA | HSA, CD5, CD68, HMW-CK |
| 16 | Biopsy | N/A | CK7, CK8, CK19, vimentin, CEA | HSA |
| 17 | Biopsy | N/A | CK7, CK8, CK19, vimentin, CD34 | HSA, CEA, HMW-CK |
| 18 | Biopsy | N/A | CK19, vimentin, CEA, p53 | CD31, CD34 |
| 19 | Biopsy | N/A | CK19, vimentin, CEA | CK7, Desmin, HSA, SMA, c-kit, S-100 |
| 20 | Biopsy | N/A | CK, CK19, vimentin, CEA | HSA, CD31 |
| 21 | Biopsy | N/A | CK7, CK19, vimentin, MUC1 | HSA, CD10 |
| 22 | Biopsy | A malignant tumor with  a carcinomatous and a sarcomatous component | CK7, vimentin | CK20, HepPar1 |
| 23 | Biopsy,  autopsy | Perferation of atypical fibrohistioytic spindle or giant cell | KER, EMA, vimentin, CEA | AFP, S-100, AAT |
| 24 | Autopsy | Poorly differentiated adenocarcinoma | Low molecular cytokeratin, vimentin | UEA-1, desmin |
| 25 | Biopsy | Favor sarcomatous ICC | N/A | N/A |
| 26 | Resection | Sarcomatous ICC | N/A | N/A |
| 27 | Resection | Sarcomatous ICC | N/A | N/A |
| 28 | Biopsy | Sarcomatous ICC | N/A | N/A |
| 29 | Biopsy | Favor sarcomatous ICC | N/A | N/A |
| 30 | Resection | Sarcomatous ICC | N/A | N/A |
| 31 | Resection | Sarcomatous ICC | N/A | N/A |
| 32 | Resection | Sarcomatous ICC | N/A | N/A |
| 33 | Biopsy | Favor sarcomatous ICC | N/A | N/A |
| 34 | Resection | Sarcomatous ICC | N/A | N/A |
| 35 | Biopsy | Sarcomatous ICC | N/A | N/A |
| 36 | Resection | Sarcomatous ICC | N/A | N/A |
| 37 | Resection | Sarcomatous ICC | N/A | N/A |
| 38 | Resection | Sarcomatous ICC | N/A | N/A |
| 39 | Resection | Sarcomatous ICC | N/A | N/A |
| 40 | Resection | Sarcomatous ICC | N/A | N/A |
| 41 | Resection | Sarcomatous ICC | N/A | N/A |
| 42 | Resection | Sarcomatous ICC | N/A | N/A |
| 43 | Resection | Sarcomatous ICC | N/A | N/A |
| 44 | Resection | Sarcomatous ICC | N/A | N/A |
| 45 | Resection | Sarcomatous carcinoma | N/A | N/A |
| 46 | Biopsy | Sarcomatous carcinoma | N/A | N/A |
| 47 | Autopsy | Tubular adenocarcinoma  with a fibrous stroma, consistent with cholangiocarcinoma, and sarcomatous carcinoma | vimentin | S-100, desmin, AFP, albumin, myoglobin |
| 48 | Resection | sarcomatous ICC | CK, vimentin | N/A |
| 49 | N/A | Moderately differentiated adenocarcinoma | KER, EMA, CA19-9 | PAS, CEA, AFP, vimentin, actin, desmin, S-100,NSE |
| 50 | Resection | Moderately differentiated adenocarcinoma | KER, EMA, vimentin | PAS, CEA, AFP, CA199, actin, desmin, S-100, NSE |
| 51 | N/A | Moderately differentiated adenocarcinoma | None | PAS, CEA, AFP, CA199, actin, desmin, S-100,  NSE, KER, EMA, vimentin |
| 52 | N/A | Moderately differentiated adenocarcinoma | PAS, KER, EMA, vimentin | CEA, CA199, AFP, actin, desmin, S-100, NSE |
| 53 | N/A | Poorly differentiated adenocarcinoma | KER, EMA | PAS, CEA, AFP, CA199, actin, desmin, S-100, NSE, vimentin |
| 54 | N/A | Poorly differentiated adenocarcinoma | PAS, KER, EMA, CEA | vimentin, CA199, AFP, actin, desmin, S-100, NSE |
| 55 | Resection | Poorly differentiated adenocarcinoma | None | PAS, CEA, AFP, CA199, actin, desmin, S-100,  NSE, KER, EMA, vimentin |
| 56 | Resection | Choiangiocarcinoma with sarcomatous transformation. | wide-spectrum keratin, vimentin, CEA | muscle actin, AAT, S-100, AFP |
| 57 | Resection | Moderately differentiated tubular adenocarcinoma | KER, EMA, vimentin | AFP, CEA, CA199, actin, desmin, S-100 |
| 58 | Resection | Intrahepatic cholangiocarcinoma with malignant fibrous histiocytoma-like sarcomatous change | AAT, vimentin, F13a | desmin, EMA, CYT, SMA, CEA, AFP |
| 59 | Autopsy | Moderately differentiated tubular adenocarcinoma and round cell | CK19, vimentin, CD44s | b-catenin |
| 60 | Resection | cholangiocarcinoma with sarcomatous change | N/A | N/A |
| 61 | Resection | Sarcomatous ICC | AE1/AE3, STAT6, SOX10, CD34, CK19, desmin, MUC1, vimentin, SMA, S-100 | N/A |
| 62 | Resection | Sarcomatoid intrahepatic  cholangiocarcinoma | CK-pan, CK8, vimentin | CK7, CK20, HepPar-1 |
| 63 | Resection | Cholangiocarcinoma with sarcomatous changes | CK7, CK19, vimentin and keratin-1 | N/A |
| 64 | Biopsy | Sarcomatous  cholangiocarcinoma | Cytokeratin, vimentin | N/A |
| 65 | Resection | Sarcomatoid cholangiocarcinoma | EMA, CK-pan, CEA | Vimentin, S-100 |
| 66 | Resection | Sarcomatoid cholangiocarcinoma with osteoclast-like giant cells | CK19, vimentin, CD68 | Hepatocyte antigen, AFP |
| 67 | Resection | Sarcomatous intrahepatic cholangiocarcinoma | N/A | N/A |
| 68 | Resection | Sarcomatous intrahepatic cholangiocarcinoma | N/A | N/A |
| 69 | Resection | Intrahepatic less differentiated sarcomatoid cholangiocarcinoma | CD34, CK19, AE1/AE3, | CA19, hepatocytes,  AFP, HMBE-1, TTF1, CK5/6 |
| 70 | Biopsy,  autopsy | G-CSF-producing intrahepatic sarcomatoid cholangiocarcinoma | AE1/AE3, CK7, CK19,  G-CSF | Hep par-1, AFP |
| 71 | Resection | Squamous cell carcinoma | N/A | N/A |
| 72 | Autopsy | G-CSF-producing intrahepatic sarcomatoid cholangiocarcinoma | CK19, CK20, CA19-9, G-CSF, PTH-rP | antihuman hepatocyte antigen |
| 73 | Resection | G-CSF-producing intrahepatic sarcomatoid cholangiocarcinoma | G-CSF | N/A |
| 74 | Resection | Intrahepatic cholangiocarcinoma | G-CSF | N/A |
| 75 | Autopsy | Intrahepatic cholangiocarcinoma with sarcomatous change  producing G-CSF | G-CSF, vimentin, CK7, CK19, CK CAM 5.2 | Hepatocyte paraffin-1 |
| 76 | Resection | Intrahepatic sarcomatoid cholangiocarcinoma | CK19, CK7, CD34 (vessels), CD31 (vessels), PD-L1, vimentin | AFP, Glypican-3,  HepPar-1, CK20,  PD-L1 |

Abbreviations: AFP: alpha-fetoprotein; CEA: carcinoembryonic antigen; CA19-9: carbohydrate antigen 19-9; CK: cytokeratin; HSA: hepatocyte specific antigen; SMA: smooth muscle actin; TTF-1: thyroid transcription factor-1; AMA: anti-mitochondria autoantibodies; UEA-1: ulex europaeus agglutinin-1; HMW-CK: high molecular weight cytokeratin; c-kit: receptor tyrosine kinase; CYT: cytochrome; HMB-45: human melanoma black 45; CD: Cluster of differentiation; PAS: periodic acid–Schiff; KER: keratin; EMA: epithelial membrane antigen; MUC1: mucin-1; AE1/AE3: pancytokeratin AE1/AE3; AE1/AE3=CK-pan; HepPar-1: hepatocyte paraffin-1; EMA: epithelial membrane antigen; G-CSF: granulocyte-colony-stimulating factor; PTH-rP: parathyroid hormone-related protein; NSE: neuron specific enolase; AAT: A-1-antitrypsin; STAT-6: signal transducer and activator of transcription 6; F13a: factor XIIIa; PD-L1: programmed cell death ligand 1; HBME-1：human bone marrow endothelial cell-1; ICC: intrahepatic cholangiocarcinoma; N/A: not available.
